# BAG3 promotes proliferation and migration of arterial smooth muscle cells by regulating STAT3 phosphorylation in diabetic vascular remodeling

**DOI:** 10.1186/s12933-024-02216-z

**Published:** 2024-04-25

**Authors:** Xinyue Huang, Jiayan Guo, Anqi Ning, Naijin Zhang, Yingxian Sun

**Affiliations:** Department of Cardiology, First Hospital of China Medical University, 155 Nanjing North Street, Heping District, Shenyang, 110001 Liaoning Province China

**Keywords:** BAG3, Diabetic vascular remodeling, VSMCs, STAT3, Phosphorylation

## Abstract

**Background:**

Diabetic vascular remodeling is the most important pathological basis of diabetic cardiovascular complications. The accumulation of advanced glycation end products (AGEs) caused by elevated blood glucose promotes the proliferation and migration of vascular smooth muscle cells (VSMCs), leading to arterial wall thickening and ultimately vascular remodeling. Therefore, the excessive proliferation and migration of VSMCs is considered as an important therapeutic target for vascular remodeling in diabetes mellitus. However, due to the lack of breakthrough in experiments, there is currently no effective treatment for the excessive proliferation and migration of VSMCs in diabetic patients. Bcl-2-associated athanogene 3 (BAG3) protein is a multifunctional protein highly expressed in skeletal muscle and myocardium. Previous research has confirmed that BAG3 can not only regulate cell survival and apoptosis, but also affect cell proliferation and migration. Since the excessive proliferation and migration of VSMCs is an important pathogenesis of vascular remodeling in diabetes, the role of BAG3 in the excessive proliferation and migration of VSMCs and its molecular mechanism deserve further investigation.

**Methods:**

In this study, *BAG3* gene was manipulated in smooth muscle to acquire *SM22αCre; BAG3*^*FL/FL*^ mice and streptozotocin (STZ) was used to simulate diabetes. Expression of proteins and aortic thickness of mice were detected by immunofluorescence, ultrasound and hematoxylin-eosin (HE) staining. Using human aorta smooth muscle cell line (HASMC), cell viability was measured by CCK-8 and proliferation was measured by colony formation experiment. Migration was detected by transwell, scratch experiments and Phalloidin staining. Western Blot was used to detect protein expression and Co-Immunoprecipitation (Co-IP) was used to detect protein interaction.

**Results:**

In diabetic vascular remodeling, AGEs could promote the interaction between BAG3 and signal transducer and activator of transcription 3 (STAT3), leading to the enhanced interaction between STAT3 and Janus kinase 2 (JAK2) and reduced interaction between STAT3 and extracellular signal-regulated kinase 1/2 (ERK1/2), resulting in accumulated p-STAT3(705) and reduced p-STAT3(727). Subsequently, the expression of matrix metallopeptidase 2 (MMP2) is upregulated, thus promoting the migration of VSMCs.

**Conclusions:**

BAG3 upregulates the expression of MMP2 by increasing p-STAT3(705) and decreasing p-STAT3(727) levels, thereby promoting vascular remodeling in diabetes. This provides a new orientation for the prevention and treatment of diabetic vascular remodeling.

**Supplementary Information:**

The online version contains supplementary material available at 10.1186/s12933-024-02216-z.

## Introduction

Chronic hyperglycemia can directly induce the occurrence and development of cardiovascular complications of diabetes through multiple mechanisms, such as inflammation, oxidative stress and increased formation of AGEs. Diabetic vascular remodeling is an extremely important pathological mechanism of diabetic cardiovascular complications and the major cause of death in diabetic patients, which is mainly manifested by excessive proliferation and migration of VSMCs leading to arterial wall thickening and eventually vascular remodeling [1].

VSMCs are highly plastic and exhibit different phenotypes in response to environmental stimuli, thus exercising a variety of functions [2]. Many previous experiments and clinical studies have focused on the changes in the function of VSMCs under the condition of high glucose, and how to reduce diabetic vascular remodeling by improving the function of VSMCs [3, 4]. However, due to the lack of breakthrough in experiments, there is no universal clinical treatment for alleviating diabetic vascular remodeling at present.

Bcl-2-associated athanogene (BAG) family proteins are involved in regulating important biological processes such as autophagy, apoptosis and protein homeostasis through interacting with Bcl-2 family proteins [5]. As early as 1997, scientists discovered that BAG1 was involved in regulating the activity of heat shock protein 70 (Hsp70) [6]. Subsequently, the role of BAG family proteins in various biochemical events has been gradually confirmed [5, 7, 8]. Among them, BAG3 protein is proved to mediate various biological processes such as autophagy, apoptosis and proliferation by binding various chaperone proteins [7].

BAG3 is highly expressed in skeletal muscle, myocardium and various tumor cells, and its expression can also be induced by stress [9, 10]. It can bind to Hsp70 and Bcl-2 through the BAG domain and interact with a variety of other proteins through the WW domain, proline-rich (PXXP) repeats and IPV(Ile-Pro-Val) motifs [11–20]. Besides, BAG3 is known to bridge many important crosstalk, thereby activating the ubiquitin-proteasome system and the selective autophagy system [21]. In addition, relevant studies have confirmed that BAG3 not only has the ability to maintain cell survival and inhibit apoptosis, but also can promote cell proliferation and migration [22–25]. As the excessive proliferation and migration of VSMCs is crucial in the occurrence and development of diabetic vascular remodeling, the role of BAG3 in diabetic vascular remodeling and its molecular mechanism are worthy of further investigation.

## Materials and methods

### Diabetes modeling in transgenic mice

The transgenic mice in this experiment were ordered from Shanghai Model Organisms Center, Inc. *BAG3* gene were specific knockout in smooth muscle using specific knockout technique in C57BL/6 background mice. The mice were kept in a specific pathogen-free animal laboratory provided by China Medical University. All animal experiments in this study were approved by the Animal Science Committee of China Medical University, and the approval number was TZ2022024. All procedures in the research conform to the guidelines from Directive 2010/63/EU of the European Parliament on the protection of animals used for scientific purposes or the NIH Guide for the Care and Use of Laboratory Animals.

As estrogen may interfere with the experimental results [26–30], the mice for modeling and experiments were 6-week-old male with a weight of 20–25 g. In this study, STZ was injected intraperitoneally to induce diabetes in mice for 2 consecutive days at a dose of 100 mg/kg per day, while the control group was injected intraperitoneally with the same volume of sodium citrate. After 2 days, if the blood glucose in the tail vein of mice was more than or equal to 16.7mmol/L, the model of diabetes in mice was considered to be successful. After successful modeling, the mice were kept for 6 weeks and the blood glucose in the tail vein was measured twice a week. After 6 weeks, the modeling was finished and the following experiments began.

### Ultrasound examination of mice aorta

Hair removal was performed on each group of mice the day before ultrasound examination. On the day of the ultrasound examination, the mice were anesthetized by mask after induction anesthesia using the anesthesia induction box. Subsequently, aorta of mice was examined using vinno6lab ultrasonic instrument and X10-23 L probe.

### Preparation of frozen sections

The aorta was fixed with 4% paraformaldehyde, followed by 5%, 10%, 15%, 20%, 30% sucrose solution. Subsequently, the aorta was removed from 30% sucrose solution, placed in an embedding mold and infiltrated with optimal cutting temperature compound. The mold was then placed in liquid nitrogen and the aorta was frozen. Afterwards, the samples were cut into 10 μm slices using a freezing microtome, and the slides were placed face up at room temperature overnight.

### HE staining

After soaked in hematoxylin for 5–8 min, the slides were cleaned and then immersed in 1% hydrochloric ethanol for a few seconds before cleaned again. After soaking in eosin staining solution for 2 min, the slides were cleaned and dehydrated in the following order: 95% ethanol I, 2 min; 95% ethanol II, 5 min; 100% ethanol I, 3 min; 100% ethanol II, 5 min; Xylene I, 5–10 min; Xylene II, 5 min. After dehydration, the slides were taken out, dried and sealed with neutral balsam for observation and photography.

### Mice aorta immunofluorescence

After the slides were perforated for 15 min using 0.5% Triton dissolved in PBS, they were cleaned, dried and sealed with 5% goat serum dissolved in PBS for 1 h. A primary antibody formulated with 0.5 ml PBS: 0.5 ml PBS dissolved in 0.5% Triton: 10ul goat serum: 10ul BAG3 antibody was then incubated overnight. On the next day, after the primary antibody was cleaned, the fluorescent secondary antibody was prepared with PBS at a ratio of 1:200 avoiding light. The secondary antibody was cleaned after incubation at room temperature for 1 h, and DAPI staining was used for 5 min. After staining, the slides were cleaned and sealed with AntiFade Mounting Medium and then observed and photographed under a fluorescence microscope.

### Cell culture

The HASMC used in this study was purchased from American Type Culture Collection and was then cultured in a cell incubator at 37℃ with 5% CO_2_. Low-glucose Dulbecco’s modified eagle medium (DMEM) containing 10% fetal bovine serum was used for routine culture. If cells needed to be starved in advance, the medium was changed to low-glucose DMEM without fetal bovine serum 24 h before the start of the experiments.

### Overexpression of target protein

All plasmids in this research were purchased from Shanghai Genechem Co., Ltd. Transfection was performed when cells were cultured to a density of about 70%. The transfection solution for each 6 cm petri dish was prepared as follows: 500 µl PBS, 4 µg plasmid and 10 µl Higene transfection reagent. The transfection solution was incubated at room temperature for 15 min and then added into the cells. The medium was replaced 8 h later, and cells were collected 48 h later for following experiments.

### Downregulation of target protein

The siRNA of BAG3 used in this research (stB0003929A/genOFFTM st-h-BAG3_001) was purchased from Guangzhou RiboBio Co., Ltd. The working solution for each 6 cm petri dish was prepared as follows: 500 µl Jet Buffer, 10 µl siRNA and 11 µl JetPrime reagent. The working solution was incubated at room temperature for 15 min and then added into the cells after the medium was replaced with serum-free low-glucose DMEM. After 24 h, the medium was replaced with low-glucose DMEM containing 10% fetal bovine serum. After 72 h, the cells were collected for following experiments.

### Cell viability detection by CCK-8

In this study, CCK-8 was used to analyze the viability of HASMC. When measuring cell viability, the medium was changed to 100 µl CCK-8 working solution. The ratio of complete medium to CCK-8 in CCK-8 working solution was 10 to 1. After 2 h, the 96-well plate was put into the microplate reader to obtain the absorbance value of each well at 450 nm wavelength. Finally, Graphpad Prism was used to process the data.

### 10 transwell migration experiment

Corning Transwell cell culture dishes were used to detect the migration of HASMC. After starvation for 24 h, cells were re-suspended in serum-free low-glucose DMEM, and the concentration of cells was adjusted to about 3 × 10^5^/ml. 800 µl low-glucose DMEM containing 10% fetal bovine serum was added into the lower chambers of 24-well plate. Subsequently, 100 µl cell suspension was added into the upper chambers. After 24 h, the cells were cleaned with PBS and fixed with 4% paraformaldehyde. After fixation, the cells were washed and stained with 0.1% crystal violet. After staining, the cells were washed and the upper layer of unmigrated cells were cleaned. Finally, migrated cells were observed and photographed under an inverted microscope.

### 11 scratch experiment

The experiment was started when 100% fusion of HASMC was observed in the 6-well plate. A 20 µl pipettor was used to mark parallel lines in the growing area of HASMC, and the cells were cleaned with PBS. Serum-free low-glucose DMEM was added to the culture dish, and then the culture continued. The 6-well plate was observed and photographed under a microscope at appropriate times.

### 12 Colony formation experiment

Each well of the 6-well plate was inoculated with 2000 HASMC, and the experiment ended 7 days later. The cells in the BAG3 overexpression and siBAG3 groups were transfected every other day, and AGEs was added each time the medium was changed. After 7 days, the 6-well plate was cleaned with PBS and fixed with 4% paraformaldehyde. After fixation, the 6-well plate was cleaned with PBS and added 0.1% crystal violet for staining. After staining, the 6-well plate was washed and pictures were taken.

### 13 phalloidin staining

Slides were placed in a 24-well plate, and then 3 × 10^4^ HASMC were inoculated in each well. When staining is required, clean the plate and fix the cells with 4% paraformaldehyde. Afterwards, clean the plate and add PBS containing 0.5% Triton X-100 for 15 min, then wash the plate and add PBS containing 5% BSA for 30 min. Phalloidin and PBS containing 0.5% Triton X-100 were mixed at a ratio of 1:200. After staining for 1 h avoiding light, clean the plate and add DAPI for 2 min. Subsequently, the slides were taken out, fixed with AntiFade Mounting Medium, observed and photographed under a fluorescence microscope.

### 14 western blot

Tissues and cells were lysed with lysis buffer (10 mM NaF, 137 mM NaCl, 50mM Tris-HCl (pH 7.6), 0.1 mM Na3VO4, 1 mM EDTA, 10% glycerol, 1% Nonidet P-40 (NP-40), 1mM PMSF, protease and phosphatase inhibitors). The protein samples were quantified, and the total quality and volume of each protein samples were adjusted to be the same according to the target protein expression. The SDS-PAGE gels required for electrophoresis in this research were 8% and 10%. 5 µl PageRuler Protein Ladder was added and then samples were added into the lanes. After electrophoresis, the proteins were transferred to PVDF membranes and then placed in 5% BSA solution for 1 h. Afterwards, PVDF membranes were incubated in primary antibodies overnight. PVDF membranes were removed the next day, cleaned with TBST solution and incubated in the second antibodies for 2 h. Upon completion of incubation, luminescence was obtained after PVDF membranes were cleaned with TBST solution.

### 15 Co-IP

The protein samples were quantified to ensure that the total protein quality was 2 mg and the sample volume was 800 µl. Then, after 2 µl primary antibodies were added and mixed for 3 h, 35 µl Protein A/G Beads were added and mixed at 4℃. After overnight, the Beads were cleaned, mixed with Loading Buffer and heated at 100℃ for 7 min, then electrophoresis of the supernatant was performed. Meanwhile, Western Blot was performed with the same protein samples as reference for subsequent analysis.

### 16 statistical analysis

Western Blot, Transwell, scratch experiment, phalloidin staining and colony formation experiment were analyzed using Adobe Illustrator 2020. Image J software v1.46 (National Institutes of Health, USA) was used for scanning of band intensities in Western Blot, colony formation experiments and Transwell. Statistical charts were made and data were analyzed using Graphpad Prism (Version 8.0). Western Blot in this study were all repeated for at least three times. After quantization of the results, continuous variables such as gray values were expressed by mean ± standard deviation (SD), in which Tubulin or GAPDH were used to standardize the band intensities in Western Blot. All data were evaluated for variance homogeneity using the F test and Brown-Forsythe test, and for normality using the Shapiro-Wilk test, followed by the corresponding one-way ANOVA or two-way ANOVA, and the Bonferroni method was used for post hoc test.

### 17 antibodies and reagents

Rabbit-anti-BAG3 antibody (10599-1-AP, Proteintech, WB: 1:2000; IP: 1:1000; IF: 1:100), rabbit-anti-MMP2 antibody (10373-2-AP, Proteintech, WB: 1:1000), rabbit-anti-MMP9 antibody (10375-2-AP, Proteintech, WB: 1:1000), rabbit-anti-PCNA antibody (10205-2-AP, Proteintech, WB: 1:1000), mouse-anti-Tubulin antibody (66031-1-Ig, Proteintech, WB: 1:1000), mouse-anti-GAPDH antibody (60004-1-Ig, Proteintech, WB: 1:1000), mouse-anti-Flag antibody (66008-4-Ig, Proteintech, WB: 1:1000; IP: 1:1000), rabbit-anti-JAK2 antibody (3230 S, CST, WB: 1:1000), rabbit-anti-p-JAK2 antibody (3776 S, CST, WB: 1:1000), mouse-anti-STAT3 antibody (9139 S, CST, WB: 1:1000; IP: 1:1000), rabbit-anti-p-STAT3(705) antibody (9145 S, CST, WB: 1:1000; IF: 1:100), rabbit-anti-p-STAT3(727) antibody (94,994 S, CST, WB: 1:1000; IF: 1:100), mouse-anti-ERK1/2 antibody (sc-514,302, Santa, WB: 1:1000), mouse-anti-p-ERK1/2 antibody (sc-81,492, Santa, WB: 1:1000), mouse-anti-GATA3 antibody (66400-1-Ig, Proteintech, WB: 1:1000), HRP goat-anti-rabbit IgG antibody (A21020, Abbkine, WB: 1:10000), HRP goat-anti-mouse IgG antibody (A21010, Abbkine, WB: 1:10000), Rhodamine(TRITIC) conjugated goat-anti-rabbit IgG(H + L) (SA00007-2, Proteintech, IF: 1:200), Protein A/G magnetic Beads (Cat#B23202, Biotool), AntiFade Mounting Medium (Beyotime), CCK-8 cell viability kit (B34304, Bimake), Higene transfection reagent (C1506, APPLYGEN), Jet kit (101,000,046, Polyplus), protease inhibitor (B14001, Bimake), phosphatase inhibitors (B15001, Bimake), SH-4-54 (S7337, Selleck), U0126-EtOH (S1102, Selleck).

## Results

### BAG3 promotes the proliferation and migration of VSMCs in diabetic vascular remodeling

**Fig. 1:**
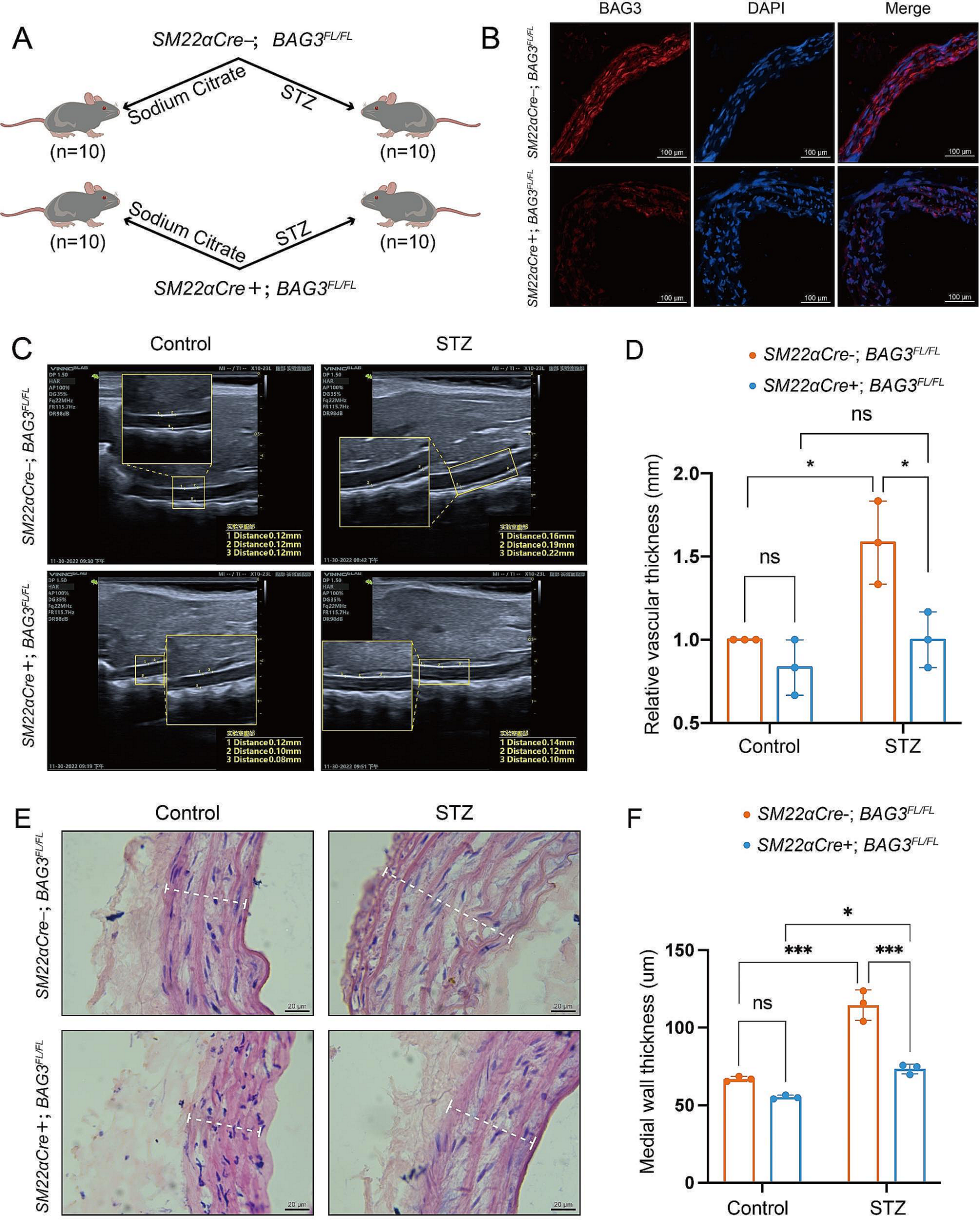
Specific knockout *BAG3* in smooth muscle inhibited the proliferation and migration of VSMCs in diabetic mice

*BAG3* gene was specifically knockout in smooth muscle of mice and the downregulation of BAG3 protein in aorta smooth muscle was confirmed by immunofluorescence and subsequently, diabetic mice were induced (Fig. 1-A-B). At the end of the modeling, aortic ultrasound confirmed that the diabetic mice expressing BAG3 underwent aortic thickening, but no aortic thickening was observed in *BAG3* specific knockout diabetic mice (Fig. 1-C-D). Meanwhile, HE staining also confirmed the above observation (Fig. 1-E-F). These results suggest that specific knockout *BAG3* in smooth muscle can inhibit the proliferation and migration of VSMCs in diabetic mice.

The top 200 genes with the lowest P-values were selected for bioinformatics analysis using GSE13760 data of GEO datasets and Pearson test. The results showed that BAG3 was closely related to the occurrence and development of diabetic vascular remodeling (Supplyment Fig. 1).

Aorta thickness was detected in *SM22αCre-;BAG3*^*FL/FL*^ control group, *SM22αCre-;BAG3*^*FL/FL*^ diabetes group, *SM22αCre+;BAG3*^*FL/FL*^ control group and *SM22αCre+;BAG3*^*FL/FL*^ diabetes group (*n* = 10). A, grouping and modeling of mice. B, BAG3 protein expression in mice aorta was detected by immunofluorescence. C-D, aortic thickness of mice was detected by vascular ultrasound and expressed by mean ± SD. Data were evaluated by two-way ANOVA, and the Bonferroni method was used for post hoc test. *N* = 3 mice/subgroup. E-F, aortic thickness of mice was detected by HE staining and expressed by mean ± SD. Data were evaluated by two-way ANOVA, and the Bonferroni method was used for post hoc test. *N* = 3 mice/subgroup. *** is *p* < 0.001, * is *p* < 0.05.

### BAG3 is involved in the proliferation and migration of AGEs-induced HASMC

#### Induction of AGEs on BAG3 expression and HASMC proliferation and migration is concentration-dependent

**Fig. 2:**
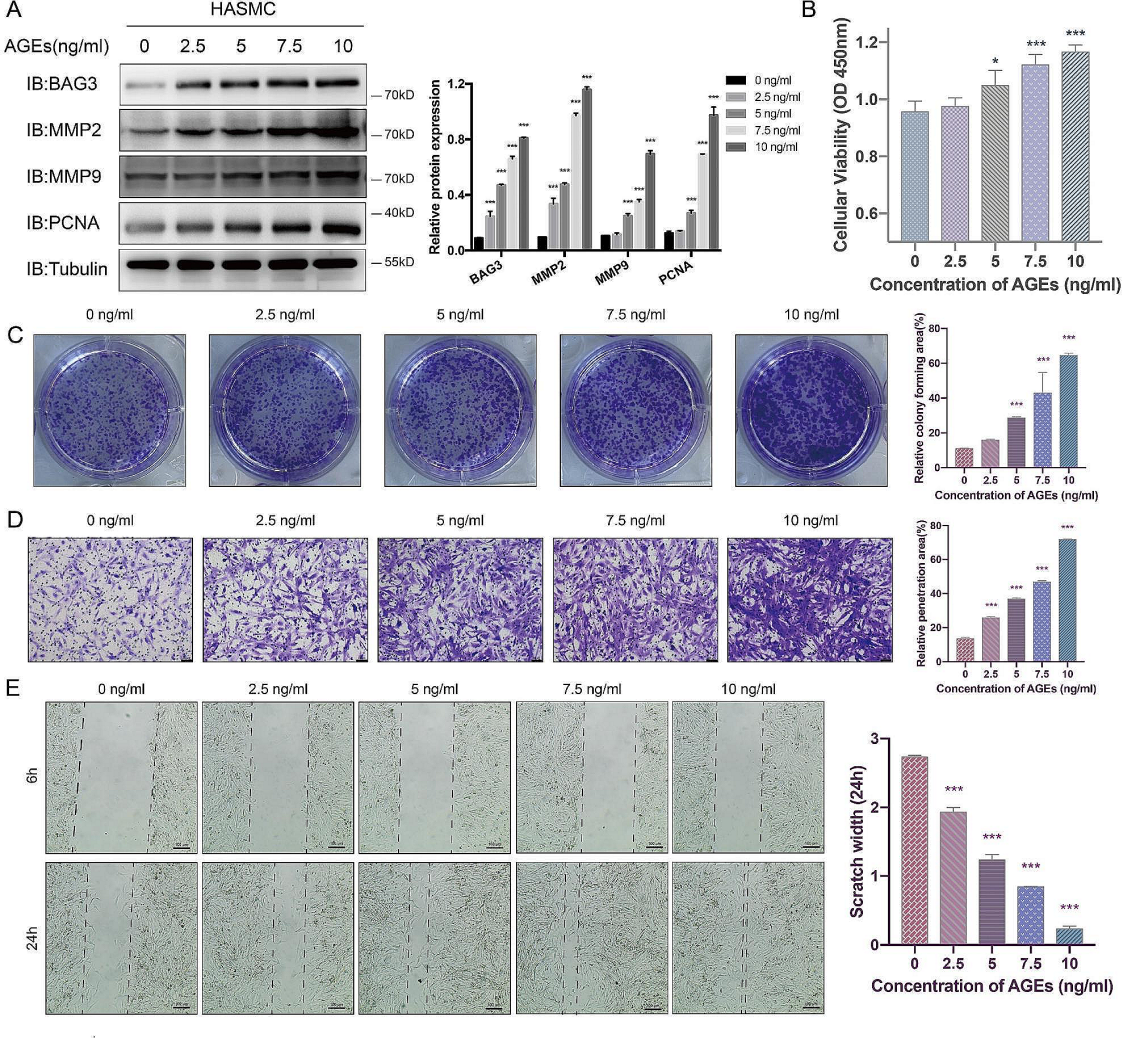
Induction of AGEs on BAG3 expression and HASMC proliferation and migration is concentration-dependent

Western Blot showed that the expression of BAG3, MMP2, MMP9 and PCNA protein in HASMC increased with AGEs concentration (Fig. 2-A). CCK-8 and colony formation experiments indicated that the viability and proliferation of HASMC gradually increased with AGEs concentration (Fig. 2-B-C). The scratch and Transwell experiments confirmed that the migration of HASMC increased with AGEs concentration as well (Fig. 2-D-E). These results suggest that AGEs induce BAG3 expression and proliferation and migration of HASMC.

Protein expression and cell function were detected after HASMC were treated with different concentrations of AGEs for 24 h. A, the expression of various proteins was detected by Western Blot, and Image J was used for densitometric scanning of band intensities. B, the viability of HASMC was detected by CCK-8 experiment. C, colony formation experiments were conducted and scanned by Image J to evaluate HASMC proliferation. D, Transwell experiments were conducted and scanned by Image J to detect HASMC migration. E, HASMC migration was detected by scratch experiments and measured by AI. Experiments in this study were all repeated for at least three times. All data were expressed by mean ± SD and evaluated by one-way ANOVA, and the Bonferroni method was used for post hoc test. *** is *p* < 0.001, * is *p* < 0.05 compared with control group without AGEs stimulation.

#### Upregulation of BAG3 promotes the proliferation and migration of AGEs-induced HASMC

**Fig. 3:**
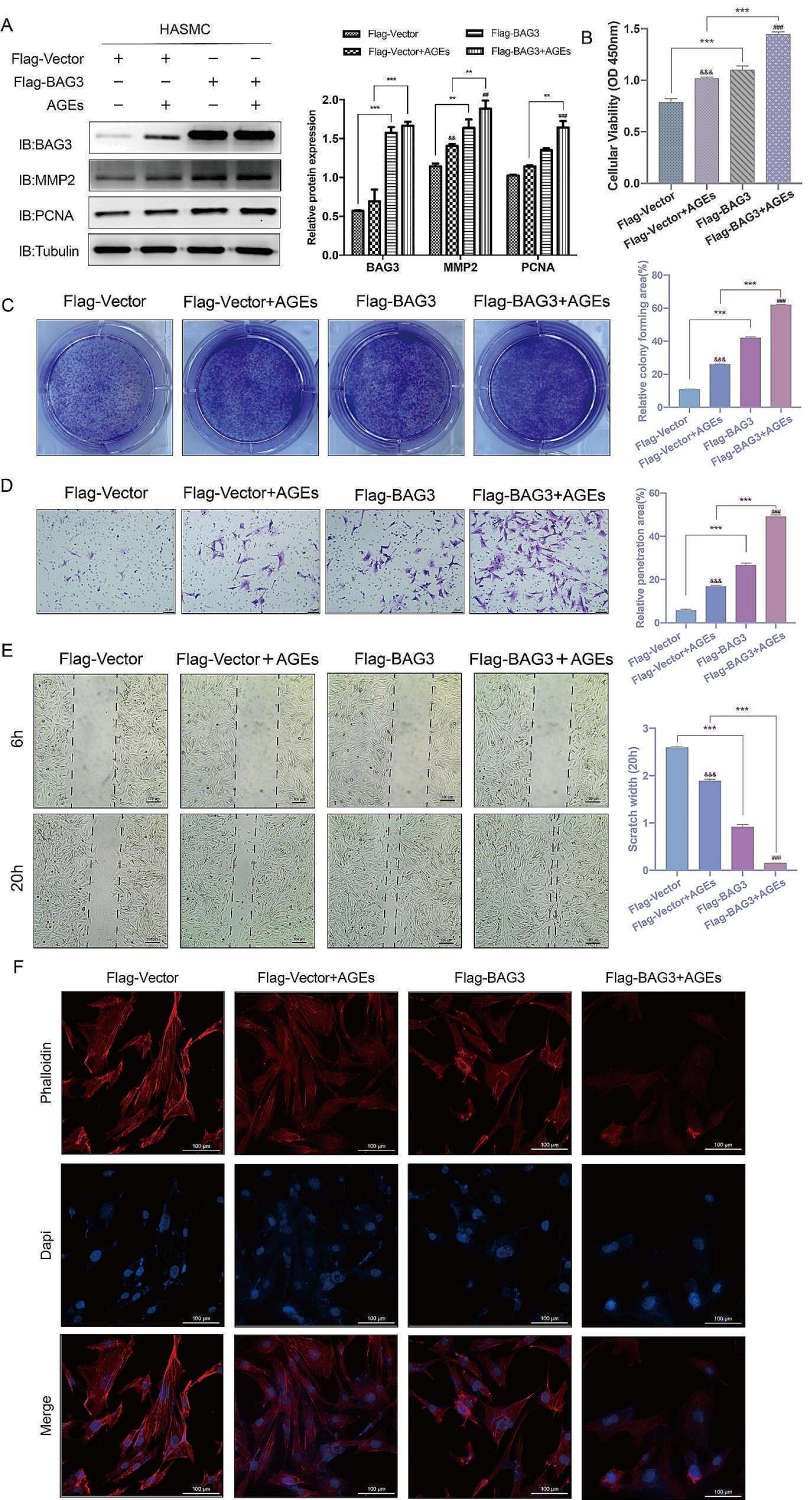
Upregulation of BAG3 promotes the proliferation and migration of AGEs-induced HASMC

After transfection of Flag-Vector or Flag-BAG3 plasmid, 10ng/ml AGEs were used to treat HASMC for 24 h. Western Blot showed that overexpression of BAG3 further upregulated the expression of MMP2 and PCNA protein stimulated by AGEs (Fig. 3-A). CCK-8 and colony formation experiments indicated that the proliferation of HASMC was enhanced after stimulation of AGEs, and was further promoted by overexpression of BAG3 (Fig. 3-B-C). Meanwhile, the scratch and Transwell experiments showed that the migration of HASMC was enhanced after AGEs stimulation, and was further promoted by overexpressing BAG3 (Fig. 3-D-E). Since the scratch observed under the microscope was about to vanish at 20 h, the experiment was ended at this time point. In addition, phalloidin staining confirmed that exogenous overexpression of BAG3 further decreased the cytoskeletal density of HASMC under AGEs stimulation, suggesting that BAG3 could promote the transition of HASMC into synthetic phenotype dominated by proliferation and migration, instead of maintaining contractile phenotype (Fig. 3-F). These results suggest that upregulation of BAG3 promotes the proliferation and migration of AGEs-induced HASMC.

After transfection of Flag-Vector or Flag-BAG3 plasmid, 10ng/ml AGEs were added for 24 h and then protein expression and cell function were assessed. A, the expression of various proteins was detected by Western Blot, and Image J was used for densitometric scanning of band intensities. B, HASMC viability was detected by CCK-8 experiments. C, colony formation experiments were conducted and scanned by Image J to evaluate HASMC proliferation. D, Transwell experiments were conducted and scanned by Image J to detect HASMC migration. E, HASMC migration was detected by scratch experiments and measured by AI. F, the cytoskeletal structure of HASMC was observed by phalloidin staining. Experiments in this study were all repeated for at least three times. Data were expressed by mean ± SD and evaluated by two-way ANOVA, and the Bonferroni method was used for post hoc test. ** is *p* < 0.01, *** is *p* < 0.001. ^&&^ is compared with Flag-Vector group *p* < 0.01, ^&&&^ is compared with Flag-Vector group *p* < 0.001. ^##^ is compared with Flag-BAG3 group *p* < 0.01, ^###^ is compared with Flag-BAG3 group *p* < 0.001.

#### Downregulation of BAG3 inhibits the proliferation and migration of AGEs-induced HASMC

**Fig. 4:**
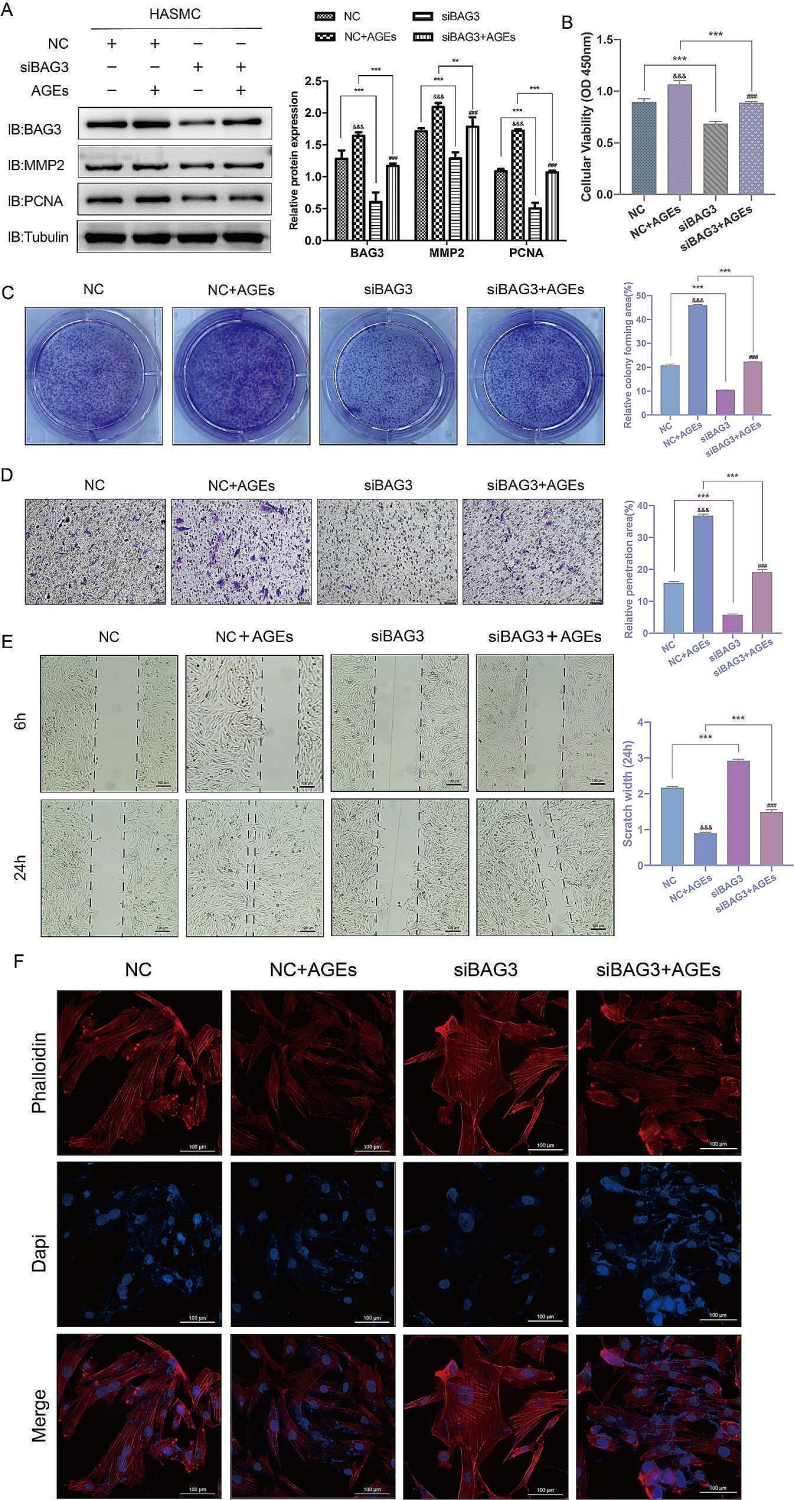
Downregulation of BAG3 inhibits the proliferation and migration of AGEs-induced HASMC

In order to further assess the impact of BAG3 on the proliferation and migration of HASMC, siBAG3 was administered and 10ng/ml AGEs were used to treat HASMC for 24 h. Western blot revealed that siBAG3 reduced the expression of MMP2 and PCNA protein stimulated by AGEs (Fig. 4-A). CCK-8 and colony formation experiments indicated that the proliferation of HASMC was enhanced after AGEs treatment, and siBAG3 inhibited the proliferation induced by AGEs (Fig. 4-B-C). Meanwhile, the scratch and Transwell experiments revealed that the migration of HASMC was enhanced after AGEs treatment, and siBAG3 reduced the migration of HASMC induced by AGEs (Fig. 4-D-E). Moreover, phalloidin staining confirmed that siBAG3 increased the cytoskeletal density under AGEs stimulation, suggesting that downregulation of BAG3 could inhibit the transition of HASMC into synthetic phenotype dominated by proliferation and migration (Fig. 4-F). Our observation implies that BAG3 downregulation inhibits the proliferation and migration of HASMC induced by AGEs.

Following the administration of siBAG3, 10ng/ml AGEs were added for 24 h and then protein expression and cell function were assessed. A, the expression of various proteins was detected by Western Blot, and Image J was used for densitometric scanning of band intensities. B, HASMC viability was detected by CCK-8 experiments. C, colony formation experiments were conducted and scanned by Image J to evaluate HASMC proliferation. D, Transwell experiments were conducted and scanned by Image J to detect HASMC migration. E, HASMC migration was detected by scratch experiments and measured by AI. F, the cytoskeletal structure of HASMC was observed by phalloidin staining. Experiments in this study were all repeated for at least three times. Data were expressed by mean ± SD and evaluated by two-way ANOVA, and the Bonferroni method was used for post hoc test. ** is *p* < 0.01, *** is *p* < 0.001. ^&&&^ is compared with NC group *p* < 0.001. ^###^ is compared with siBAG3 group *p* < 0.001.

### BAG3 promotes VSMCs migration by regulating STAT3 phosphorylation

#### Downregulation of BAG3 decreases p-STAT3(705) and increases p-STAT3(727) in mice aorta

**Fig. 5:**
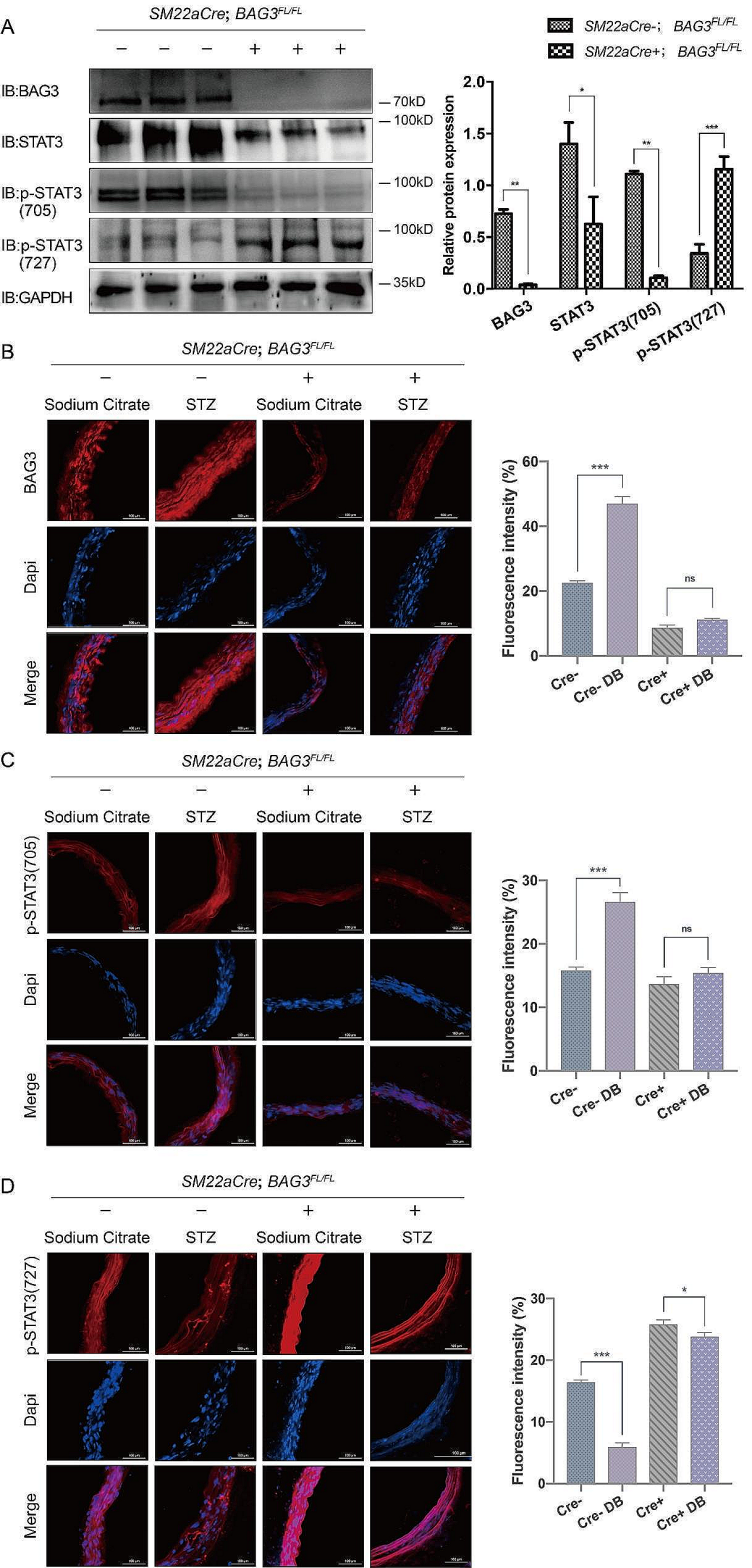
Downregulation of BAG3 affects p-STAT3(705) and p-STAT3(727)

Western Blot of *SM22αCre-;BAG3*^*FL/FL*^ and *SM22αCre+;BAG3*^*FL/FL*^ mice aorta showed that the expression of STAT3 was reduced after BAG3 downregulation. In addition, p-STAT3(705) was downregulated accompanied by p-STAT3(727) upregulation, suggesting that BAG3 was involved in regulating STAT3 phosphorylation at different sites (Fig. 5-A). Immunofluorescence staining showed that p-STAT3(705) decreased and p-STAT3(727) increased in *SM22αCre+;BAG3*^*FL/FL*^ mice aorta compared with *SM22αCre-;BAG3*^*FL/FL*^ group (Fig. 5-B-D). After downregulation of BAG3, p-STAT3(705) in mice aorta was not upregulated after diabetic modeling, while p-STAT3(727) showed a slight decrease (Fig. 5-B-D). Therefore, p-STAT3(727) may also be involved in the development of diabetic vascular remodeling through other signaling other than BAG3.

A, Western Blot was performed using aorta of *SM22αCre-;BAG3*^*FL/FL*^ and *SM22αCre+;BAG3*^*FL/FL*^ mice. *N* = 3 mice/subgroup. Data were evaluated by one-way ANOVA, and the Bonferroni method was used for post hoc test. B, BAG3 expression in mice aorta was detected by immunofluorescence. C, p-STAT3(705) in mice aorta was detected by immunofluorescence. D, p-STAT3(727) in mice aorta was detected by immunofluorescence. Experiments in this study were all repeated for at least three times. *** is *p* < 0.001, ** is *p* < 0.01, * is *p* < 0.05.

#### BAG3 regulates p-STAT3(705) and p-STAT3(727) through p-JAK2 and p-ERK1/2 respectively to promote VSMCs migration

**Fig. 6:**
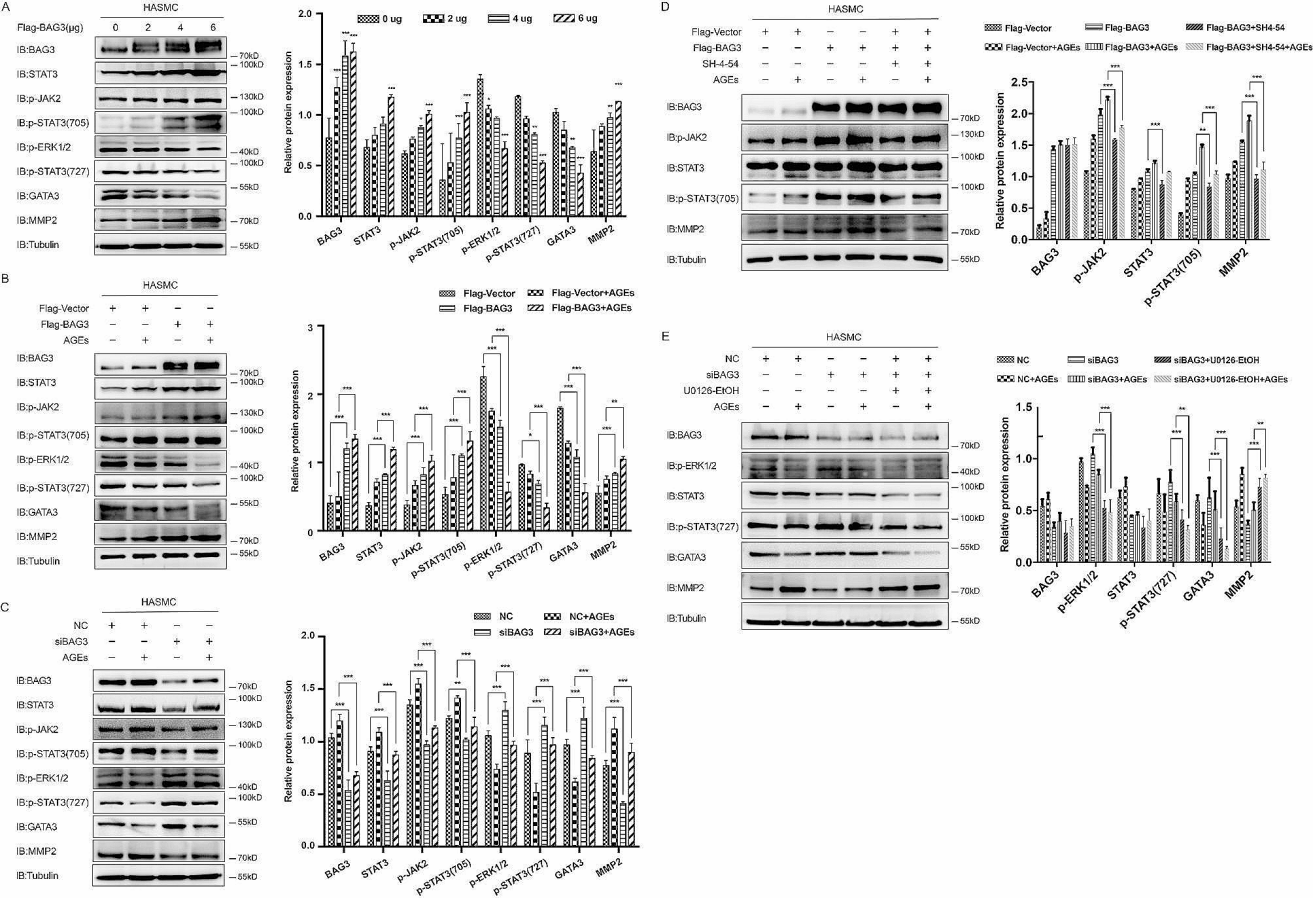
BAG3 regulates p-STAT3(705) and p-STAT3(727) through p-JAK2 and p-ERK1/2 respectively to promote VSMCs migration

In HASMC with gradient Flag-BAG3 overexpression, total STAT3 protein was increased along with the upregulation of BAG3 expression. P-JAK2 and p-STAT3(705) were increased gradually, suggesting that JAK2-STAT3 signaling was activated. On the contrary, p-ERK1/2, p-STAT3(727) and GATA binding protein 3 (GATA3) were decreased gradually, suggesting that ERK1/2-STAT3-GATA3 signaling was inhibited (Fig. 6-A). Further AGEs administration in Flag-Vector and Flag-BAG3 groups confirmed that AGEs promote the expression of BAG3, and BAG3 upregulate MMP2 expression by activating JAK2-STAT3 signaling and inhibiting ERK1/2-STAT3-GATA3 signaling, thus accelerating VSMCs migration during diabetic vascular remodeling (Fig. 6-B). Consistently, the hypothesis was proved by Western Blot after AGEs administration in the NC and siBAG3 groups (Fig. 6-C).

To clarify the role of BAG3 in VSMCs migration during diabetic vascular remodeling, 10ng/ml AGEs treatment was given to HASMC in different groups. Subsequently, p-STAT3(705) inhibitor SH-4-54 300nM or p-ERK1/2 inhibitor U0126-EtOH 20 μm was given for 24 h. Inhibition of p-STAT3(705) after BAG3 overexpression reduced MMP2 expression induced by BAG3, thereby inhibited VSMCs migration during diabetic vascular remodeling. In addition, inhibition of p-ERK1/2 after siBAG3 recovered the downregulation of MMP2 caused by siBAG3, thus promoted VSMCs migration during diabetic vascular remodeling (Fig. 6-D-E). These results further confirmed that BAG3 promotes MMP2 expression by activating JAK2-STAT3 signaling and inhibiting ERK1/2-STAT3-GATA3 signaling, thus promoting the migration of VSMCs during diabetic vascular remodeling.

A, the expression of various proteins was detected by Western Blot, and Image J was used for densitometric scanning of band intensities after gradient transfection of Flag-BAG3. Data were evaluated by one-way ANOVA, and the Bonferroni method was used for post hoc test. *** is *p* < 0.001, ** is *p* < 0.01, * is *p* < 0.05 compared with 0 ug group. B, after AGEs were administered to Flag-Vector group and Flag-BAG3 group, protein expression was detected by Western Blot. Data were evaluated by two-way ANOVA, and the Bonferroni method was used for post hoc test. C, after AGEs were administered to NC group and siBAG3 group, protein expression was detected by Western Blot. Data were evaluated by two-way ANOVA, and the Bonferroni method was used for post hoc test. D, AGEs and SH-4-54 were given to Flag-Vector and Flag-BAG3 groups, protein expression was detected by Western Blot. Data were evaluated by two-way ANOVA, and the Bonferroni method was used for post hoc test. E, AGEs and U0126-EtOH were given to NC and siBAG3 groups, protein expression was detected by Western Blot. Data were evaluated by two-way ANOVA, and the Bonferroni method was used for post hoc test. Experiments in this study were all repeated for at least three times. * is *p* < 0.05, ** is *p* < 0.01, *** is *p* < 0.001.

### BAG3 regulates STAT3 phosphorylation by interacting with STAT3 through PXXP domain

**Fig. 7:**
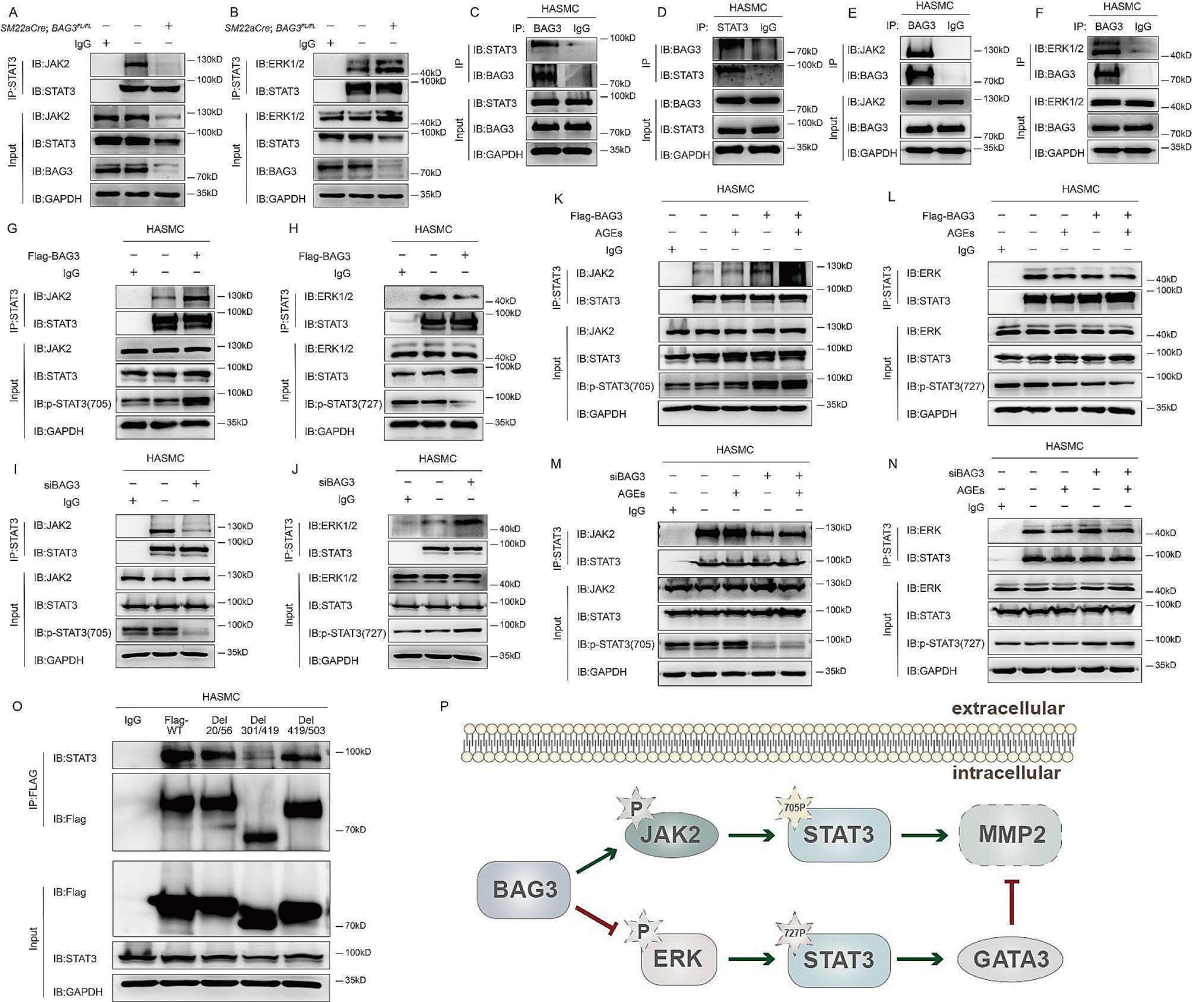
BAG3 modulates STAT3 phosphorylation by regulating the interaction of STAT3 with JAK2 and ERK1/2

Co-IP of *SM22αCre-;BAG3*^*FL/FL*^ and *SM22αCre+;BAG3*^*FL/FL*^ mice aorta showed that BAG3 deletion resulted in reduced interaction between STAT3 and JAK2 and enhanced interaction between STAT3 and ERK1/2 (Fig. 7-A-B). In HASMC, BAG3 interacts with JAK2 and STAT3, as well as with ERK1/2 and STAT3 (Fig. 7-C-F). Upregulation of BAG3 enhanced the interaction between JAK2 and STAT3, and inhibited the interaction between ERK1/2 and STAT3. Downregulation of BAG3 reduced the interaction between JAK2 and STAT3 while enhancing the interaction between ERK1/2 and STAT3 (Fig. 7-G-J). In addition, BAG3 further promoted the interaction between JAK2 and STAT3 stimulated by AGEs, and further reduced the interaction between ERK1/2 and STAT3 inhibited by AGEs (Fig. 7-K-N). Moreover, BAG3 interacted with STAT3 through its PXXP domain, sites 302–412, to regulate STAT3 phosphorylation (Fig. 7-O).

Co-IP experiments in this study were all repeated for at least three times. A-B, Co-IP was performed using aorta of *SM22αCre-;BAG3*^*FL/FL*^ and *SM22αCre+;BAG3*^*FL/FL*^ mice. C-F, in HASMC, interactions between different proteins were detected by Co-IP. G-H, after transfection of Flag-BAG3 in HASMC, interactions between different proteins were detected by Co-IP. I-J, after siBAG3 was administered in HASMC, interactions between different proteins were detected by Co-IP. K-L AGEs were given after transfection of Flag-BAG3 in HASMC, and then interactions between different proteins were detected by Co-IP. M-N, AGEs were given after administration of siBAG3 in HASMC, and interactions between different proteins were detected by Co-IP. O, interactions between BAG3 domains and STAT3 were detected by Co-IP. P, the diagram depicts that BAG3 promotes proliferation and migration of arterial smooth muscle cells by regulating STAT3 phosphorylation in diabetic vascular remodeling.

## Discussion

AGEs generated by elevated blood glucose induce the excessive proliferation of VSMCs in vascular tissues and its migration to the intima of the artery, thus leading to arterial thickening and narrowing, and finally to diabetic vascular remodeling. Although a large number of interventions for diabetic vascular remodeling have been put into clinical use, it is still one of the leading causes of death in diabetic patients. Therefore, the development of novel treatments is urgent.

As a multifunctional protein with various biological activities, BAG3 is involved in many biological processes. Previous research on BAG3 mostly focused on the correlation between BAG3 mutation and genetic diseases such as dilated cardiomyopathy [31–33]. With the incremental understanding on BAG3, its various roles as a molecular chaperone have been gradually discovered [34, 35]. In addition, numerous studies have shown that BAG3 plays an indispensable role in autophagy and cell survival by bridging multiple signaling pathways [9, 11, 12, 22]. However, because BAG3 has very important biological functions and is essential for the contraction of VSMCs, inhibition of BAG3 could cause unpredictable adverse effects in vivo, possibly leading to death [36–38], which also explained the difficulty in therapy development for BAG3 inhibitors. Therefore, it is of important clinical value to investigate the downstream molecules manipulated by BAG3 in order to alleviate the proliferation and migration of diabetic VSMCs without affecting the expression of BAG3.

Signal transducer and activator of transcription (STAT) family includes seven transcription factors, of which STAT3 participates in many biological processes such as cell proliferation, differentiation and apoptosis. Studies have confirmed that abnormally activated STAT3 can not only promote the occurrence, angiogenesis and metastasis of various tumors, but also play a crucial role in embryonic development, hematopoietic and immune system regulation [39].

Janus kinase/signal transducers and activators of transcription (JAK/STAT) are key signal pathways controlling VSMCs proliferation [40, 41]. Among them, p-JAK2, as a phosphorylase kinase, phosphorylates tyrosine at site 705 of STAT3 and leads to the proliferation of VSMCs by regulating the transcription of related genes [42]. As one of the genes positively regulated by p-STAT3(705), MMP2 is a protein that degrades extracellular matrix and is therefore often used as an indicator to observe cell migration [43–48].

Mitogen activated protein kinase (MAPK) pathways are involved in the regulation of many important cellular processes, including proliferation, differentiation, apoptosis and stress response. Ras/Raf/MAPK(MEK)/ERK pathway is the most important signal cascade in MAPK signal transduction pathway and is indispensable in maintaining cell viability [49]. As members of the MAPK family, ERK1/2 deliver extracellular signals to intracellular targets through signal cascades. In addition, p-ERK1/2 phosphorylates serine at site 727 of STAT3 [50], which subsequently promotes the expression of GATA3 [50], a transcription factor that negatively regulates the MMP2 expression [51].

SH-4-54 is a potent STAT inhibitor that acts on STAT3 at K_D_ of 300 nM [52]. Both in vivo and in vitro experiments have confirmed that SH-4-54 can effectively inhibit p-STAT3(705) and its downstream transcriptional targets, thus inhibiting the growth of glioblastoma [52]. According to our results, after SH-4-54 was administered in HASMC, the downregulation of p-STAT3(705) and its downstream molecule MMP2 were observed, indicating the inhibition of HASMC migration. It can be concluded that SH-4-54 has extensive inhibitory effects on p-STAT3(705) and its downstream molecules in different cell lines. In addition, Western Blot detected that SH-4-54 also reduced the level of p-JAK2, which was consistent with previous research [53]. In our study, considering that SH-4-54 may not only inhibit p-STAT3(705), but also affect p-STAT3(727), we detected p-STAT3(727) by Western Blot as well. The results indicated that SH-4-54 had no effect on p-STAT3(727), which was consistent with previous research [52]. As the above observation is not closely related to the main objective of this study, Western Blot of p-STAT3(727) is not presented in the manuscript. In conclusion, SH-4-54 is a potential therapeutic strategy to inhibit diabetic vascular remodeling by reducing p-STAT3(705) without affecting BAG3.

In addition, previous research on STZ-induced diabetic rats confirmed that the levels of ERK1/2 and p-ERK1/2 in testicular tissue of diabetic rats were downregulated [54], which was consistent with our observation in HASMC, namely, AGEs reduced the levels of ERK1/2 and p-ERK1/2. Subsequently, we also confirmed that the inhibitory effect of AGEs on p-ERK1/2 was reduced after BAG3 downregulation. Through literature review, we learned that BAG3 promotes the dephosphorylation of p-ERK1/2 by promoting the binding of ERK1/2 and dual specificity phosphatase 6 (DUSP6) [17]. This mechanism can further explain our observation that BAG3 promotes VSMCs migration in diabetic vascular remodeling by inhibiting ERK1/2-STAT3-GATA3 pathway.

In summary, using mice with *BAG3* gene specifically knocked out in smooth muscle, we demonstrated a novel molecular mechanism of BAG3 inducing VSMCs migration by regulating STAT3 phosphorylation at different sites, providing a new insight into the pathological basis of the occurrence and development of diabetic vascular remodeling and a new theoretical basis for future clinical drug research and development.

## Conclusions

AGEs promote the proliferation and migration of VSMCs through upregulation of BAG3. AGEs enhance the interaction between BAG3 and STAT3, while BAG3 simultaneously enhances the interaction between STAT3 and JAK2 and reduces the interaction between STAT3 and ERK1/2, leading to increased p-STAT3(705) and decreased p-STAT3(727), and subsequently positively regulates the expression of MMP2, thus promotes the migration of VSMCs.

### Electronic supplementary material

Below is the link to the electronic supplementary material.

Supplementary Material 1

## Data Availability

The authors confirm that the data supporting the findings of this study are available within the article.

## Abbreviations

AGEs: Advanced glycation end products
BAG3: Bcl-2-associated athanogene 3
Co-IP: Co-Immunoprecipitation
DMEM: Dulbecco’s modified eagle medium
ERK1/2: Extracellular signal-regulated kinase 1/2
GATA3: GATA binding protein 3
HASMC: Human aorta smooth muscle cell line
HE: Hematoxylin-eosin staining
Hsp70: Heat shock protein 70
JAK2: Janus kinase 2
MAPK: Mitogen activated protein kinase
MMP2: Matrix metallopeptidase 2
STAT3: Signal transducer and activator of transcription 3
STZ: Streptozotocin
VSMCs: Vascular smooth muscle cells
