# Supplementary figures and images for: BAG3 promotes proliferation and migration of arterial smooth muscle cells by regulating STAT3 phosphorylation in diabetic vascular remodeling
Source: Cardiovasc Diabetol. 2024 Apr 25;23:140. doi: 10.1186/s12933-024-02216-z (PMC11046803; doi:10.1186/s12933-024-02216-z)

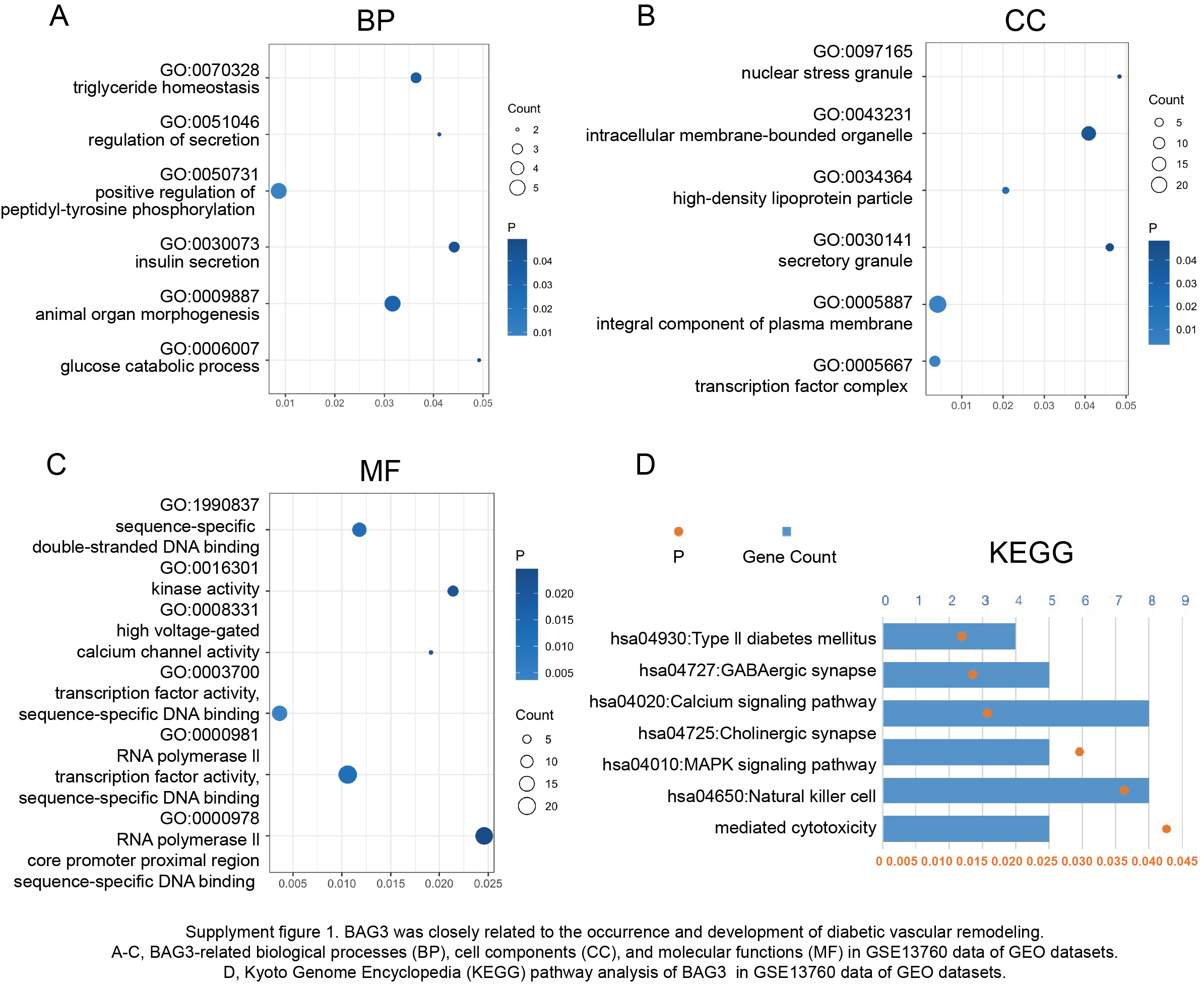

Supplement: Supplementary file 1 — Supplementary Material 1 [file 12933_2024_2216_MOESM1_ESM.png]
